# Intravascular Fasciitis of the Jugular Vein Mimicking Thrombosis and Sarcoma: A Case Report

**DOI:** 10.3389/fsurg.2021.715249

**Published:** 2021-09-27

**Authors:** Guo Xin Chen, Chu Wen Chen, Xiao Rong Wen, Bin Huang

**Affiliations:** ^1^Department of Vascular Surgery, West China Hospital, Sichuan University, Chengdu, China; ^2^Department of Ultrasound Medicine, West China Hospital, Sichuan University, Chengdu, China

**Keywords:** intravascular fasciitis, internal jugular vein, thrombosis, sarcoma, surgery

## Abstract

**Background:** Intravascular fasciitis is a rare disease that is a reactive proliferative lesion of myofibroblasts. There are rare reports that intravascular fasciitis has invaded the jugular vein as seen in this case.

**Case Presentation:** A 41-year-old female presented with right neck dull pain for 20 days. The appearance of the subcutaneous mass was oval, pink hyaline, well-demarcated, and measuring ~5 mm in diameter. Microscopically, the mass was composed of spindle cells arranged in intersecting fascicles. Immunohistochemical stains showed that the spindle cells were positive for smooth muscle actin and negative for S-100, Desmin, MyoD1, and elastin stains. The nuclei of the spindle cells were relatively uniform, and mitotic activity was observed. The overall morphological and immunohistochemical features are consistent with intravascular fasciitis.

**Conclusion:** Due to the rapid growth and vascular invasion, intravascular fasciitis created a high risk of misdiagnosing it as a sarcoma or thrombosis. Reporting this uncommon case, we raise awareness of this non-neoplastic lesion, and careful, light microscopic examination combined with immunohistochemical staining aids in the diagnosis of intravascular fasciitis.

## Introduction

Intravascular fasciitis (IVF) is a rare benign lesion arising from reactive proliferation of myofibroblasts inside the vascular lumen or in the superficial or deep fascia with involvement of small- and/or medium-size blood vessels. IVF often occurs in the upper extremities, head and neck, and lower extremities. Five cases involving the large vein are previously reported in the literature and are commonly misdiagnosed as venous thrombosis or malignant sarcoma. Here, we report a rare case of IVF that was located in the right internal jugular vein. Meanwhile, we review the literature on this rare disease.

## Case Presentation

**Figure 1:**
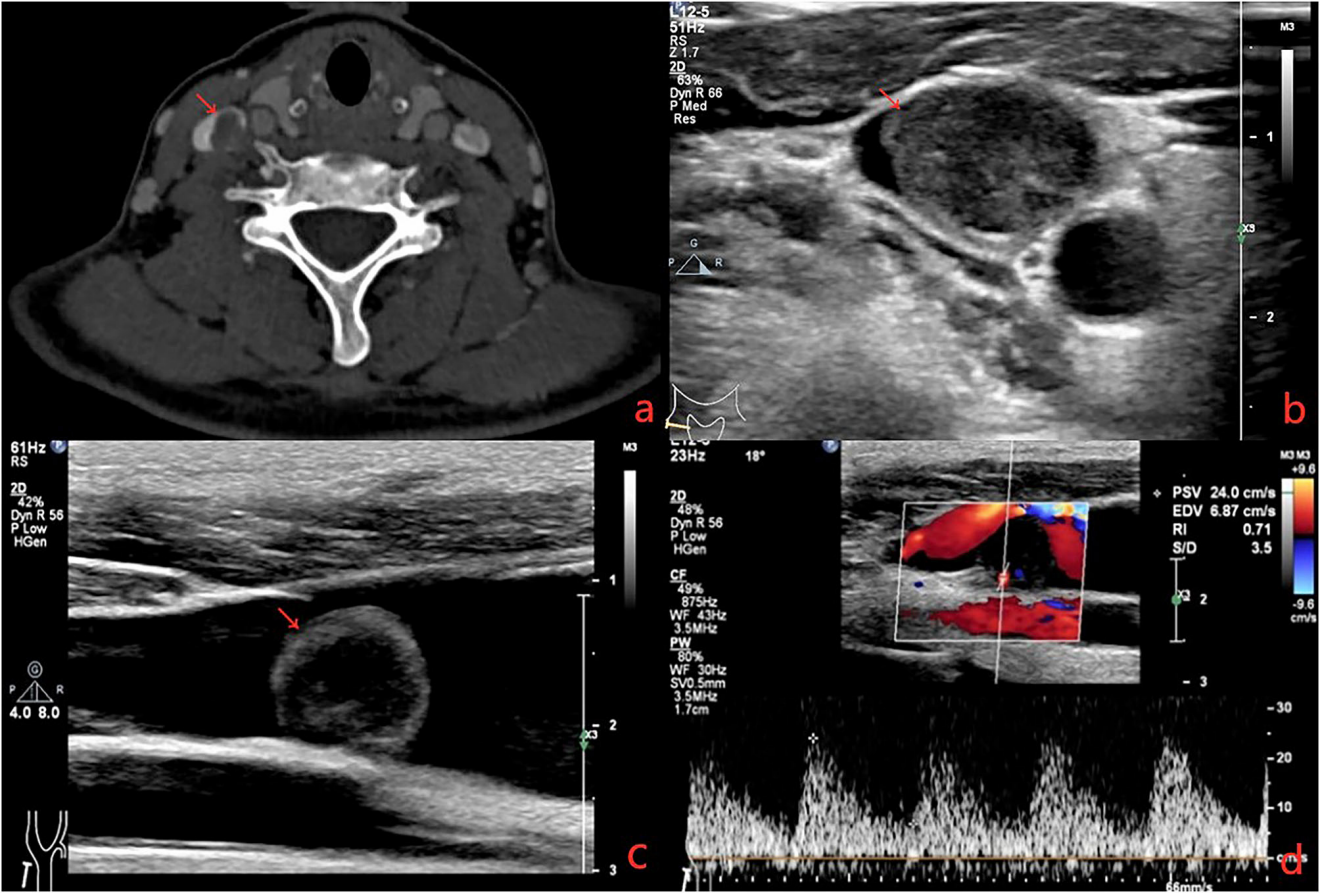
**(a)** Computerized tomography shows a solid, non-enhanced nodule in the right internal jugular vein (RJV) (red arrow: nodule, green arrow: RJV). **(b)** Ultrasound shows the nodule arising from the RJV without extravascular invasion (red arrow: nodule, green arrow: RJV). **(c)** Ultrasound shows the nodule led to severe RJV stenosis (red arrow: nodule, green arrow: RJV, red triangle: right internal carotid artery). **(d)** Doppler ultrasound detected the blood flow signals in the nodule.

**Figure 2:**
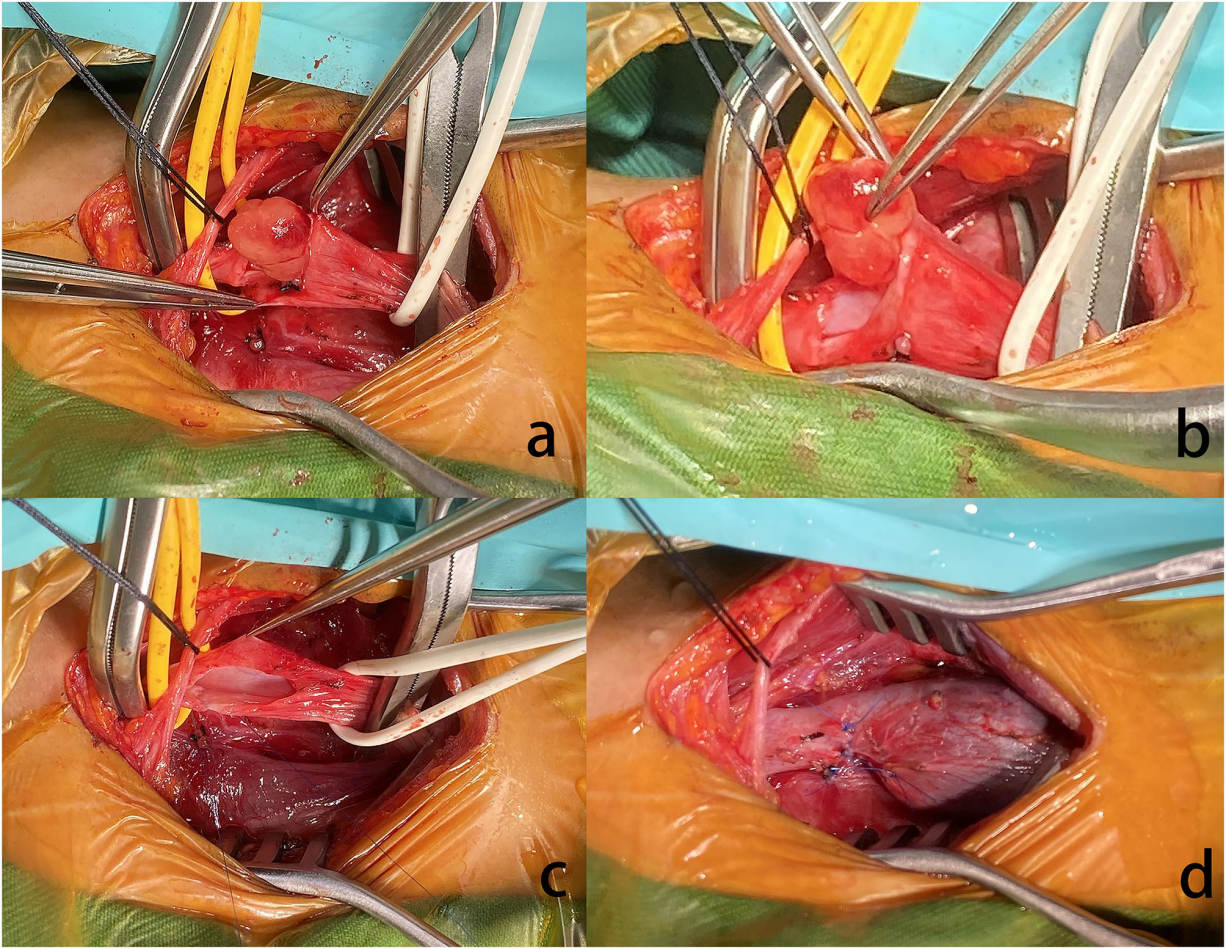
**(a)** The nodule arose from the RJV. **(b)** Grossly, the mass was pink hyaline, oval, and well-demarcated, measuring ~5 mm in diameter. **(c)** Extravascular tissues were not affected. **(d)** Due to the proximal of RJV compensatory dilation, local stenosis was observed in the RJV after reconstruction.

A 41-year-old female presented with right neck dull pain for 20 days. She denied any associated difficulty with headache, dizziness, pain, fever, or swelling. She was a nonsmoker and non-drinker and denied any drug abuse. Physical examination revealed a firm, well-defined, tender, non-mobile mass in the right neck with dimension about 10 × 10 × 10 mm. The mass did not move up or down with swallowing. Laboratory data were within normal limits. A computed tomography angiography (CTA) demonstrated the right jugular vein filling defect at the level of the cricoid cartilage–thyroid plane (Figure 1a). The grayscale ultrasound and color Doppler flow imaging showed the right internal jugular vein had a hypoechoic mass about 10 × 11 × 11 mm with a clear boundary, and the mass had a 3.9-millimeter-wide base located in the posterior wall of the internal jugular vein with a linear blood flow signal in this mass (Figures 1b,c). The pulse Doppler ultrasonography detected arterial spectrum in the mass with the peak systolic blood flow velocity: 24 cm/s, the end-diastolic blood flow velocity: 6.87 cm/s, and the resistance index: 0.71 (Figure 1d). The ultrasound indicated neoplastic lesions originating from the internal jugular vein (Figures 1b,c). After medical clearance, the patient received complete surgical excision with the internal jugular vein reconstructed under monitored anesthesia care. In the operation, the mass filled the entire lumen of the internal jugular vein, and compensated distention of the proximal part of the internal jugular vein was observed. The specimen excised was a 13 × 10 × 18 mm mass without extravascular invasion and involvement of any surrounding structures (Figure 2).

**Figure 3:**
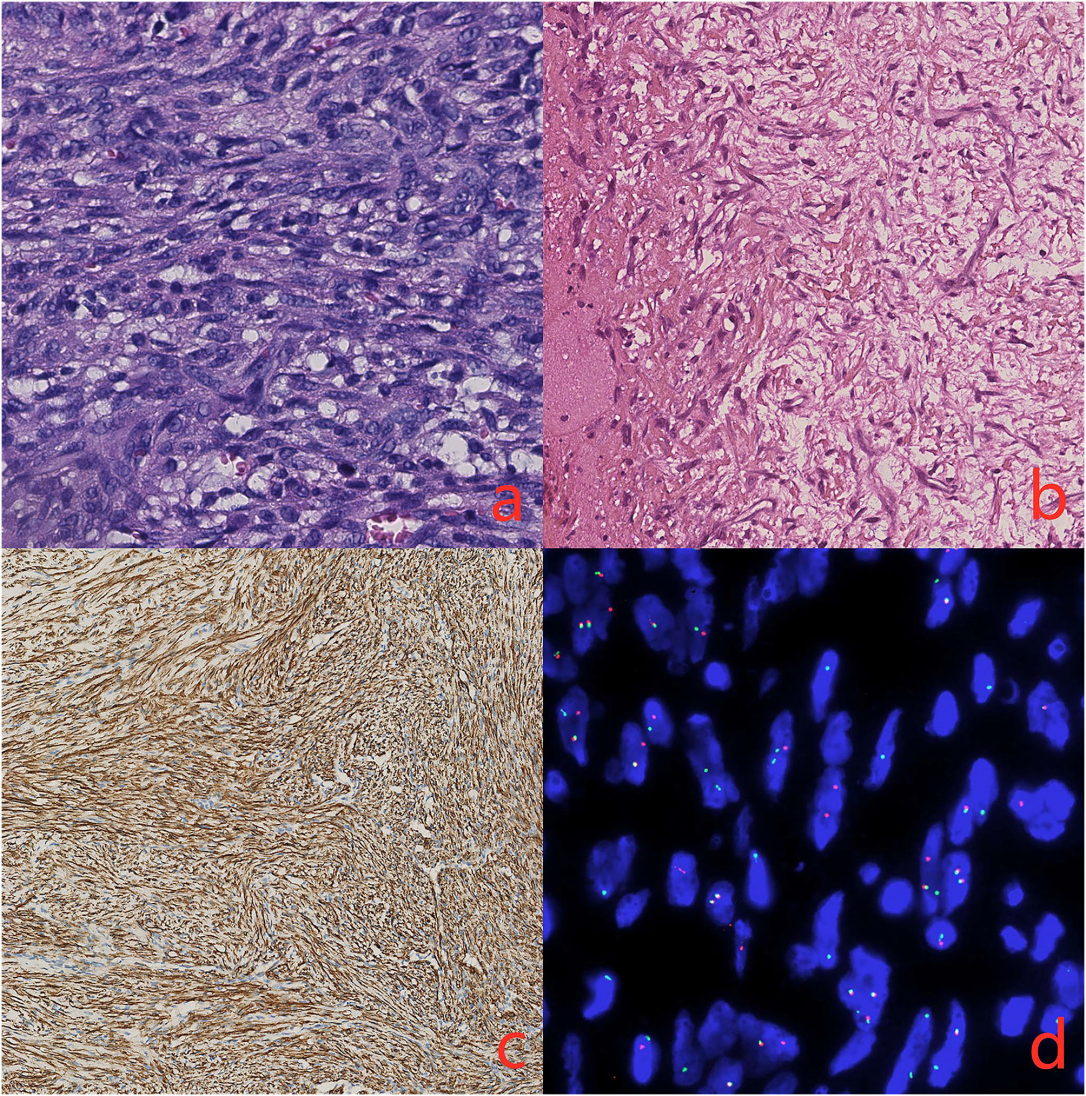
**(a)** There is a proliferation of spindle cells arranged in intersecting fascicles (hematoxylin and eosin, original magnification 40x) **(b)**. The immunohistochemical stains demonstrated elastin stains was negative (original magnification 20x) **(c)**. The spindle cells are diffusely positive for smooth muscle actin (SMA, original magnification 20x) **(d)**. FISH showing rearrangement of the USP6 locus (separation of green and red signals).

Histopathological investigation revealed that a well-circumscribed nodular growth of fibroblasts and myofibroblasts in a collagenous stroma in large areas and nuclear fission was evident. Immunohistochemically, the cells in the lesion and the blood vessel wall were diffusely positive for smooth muscle actin (SMA). S100, Desmin, MyoD1, and elastin stains were negative. Fluorescent *in situ* hybridization (FISH) showing rearrangement of the USP6 locus (separation of green and red signals) and excluding gene amplification of the MDM2 (Figure 3). Our research protocol was approved by the local ethics committee.

## Discussion

Nodular fasciitis (NF) is a benign lesion arising in the subcutaneous tissue, skeletal muscles, and fascia. IVF is a rare variant of NF, which was first described by Patchefsky and Enzinger in 1981 (1), accounting for <3% of NF (2). In total, 38 patients with IVF are reported in cases recorded in PubMed (Table 1) (1–26). So far, IVF occurring in large veins is reported in five cases (17, 18, 21, 24, 25). To our knowledge, there are no reports of intravascular fasciitis that has invaded the jugular vein as seen in this case. Possible predisposing factors of IVF are reported, including preexisting trauma, viral infection, pregnancy-related estrogen changes, and thrombosis, but the exact mechanism and causative factors remain unknown.

**Table 1:** Clinical and pathologic features of the reported cases of intravascular fasciitis.

**References**	**#**	**Age (years) and sex**	**Location**	**Symptoms**	**Gross features**	**Microscopic features**	**IHC features of spindle cells**	**Treatment**	**Follow-up**
Patchefsky and Enzinger (1)	17	20.5 (range from 0.5 to 57). 8 F and 9 M	Head and neck (*n* = 5) Upper extremity (*n* = 7) Trunk (*n* = 2) Lower extremity (*n* = 3)	No	1.5 cm^*^ (range from 0.6 to 5) single, firm, immobile	Feathery, edematous myxoid, and hyalinized background, giant cells present in 1/3 cases	N/A	CR	Lost to follow-up *n* = 8 Alive and well *n* = 7 Local recurrence *n* = 2
Freedman and Lumerman (2)	2	19, M	Right posterior mucobuccal fold	No	2.5 cm, single, firm, with ulcer	Myxoid and highly vascular background with rare mitotic figures	N/A	CR	N/A
		53, M	Left buccal mucosa	No	2.0 cm, single, firm, immobile mass	Myxoid and locally hyalinized background, no mitotic figures present	N/A	CR	N/A
Kahn et al. (3)	1	20, F	Left lower labial mucosa	No	1.5 cm, single, firm, immobile	Myxoid and highly vascular background, giant cells and mitotic figures present	N/A	CR	3 months NRM
Price et al. (4)	2	17, M	Right eye	Local swelling	2.0 cm, single	Myxoid background, giant cells and mitotic figures (<1/HPF) present	N/A	CR	36 months NRM
		20, M	Right eye	Local swelling	1.0 cm, single, firm, mobile	Myxoid background, giant cells and mitotic figures (<1/HPF) present	N/A	CR	36 months NRM
Samaratunga et al. (5)	1	49, M	Left inguinal region	Local discomfort	3.0 cm, single, firm	Myxoid background with cleft-like spaces, giant cells and mitotic figures (2/10 HPF) present	(+) Vimentin, SMA (–) LMWK, S100	CR	6 months NRM
Beer et al. (6)	1	18, F	Lateral thigh	No	2.0 cm, single, tender, mobile	Focally myxoid and highly vascular background, mitosis present	(+) SMA (–) S100	CR	24 months NRM
Sticha et al. (7)	1	4, M	Right foot	No	3.0 cm, single, firm, tender, immobile mass	Myxomatous and hyalinized background, giant cell and mitosis present	N/A	CR	24 months NRM
Ito et al. (8)	1	26, M	Right forearm	Local tenderness	Single, tender	Fibrous and vascular background, giant cells and mitotic figures (up to 1/10 HPF) present	(+) Vimentin, SMA (–) Desmin	CR	N/A
Anand et al. (9)	1	20, F	Right hand	Local swelling	3.0 cm, single, firm, mobile	Fibrous background, giant cells, and mitotic figures present	(+) SMA (–) S100, desmin	CR	12 months NRM
Sugaya and Tamaki (10)	1	66, M	Medial border of right foot	No	0.3 cm, single, mobile mass	Myxoid background, no giant cells present, rare mitotic figures	(+) Vimentin (–) S100, SMA, c-ki, Desmin, CD3, CD34, cytokeratin	CR	N/A
Pantanowitz and Duke (11)	1	17, M	Wrist	Pain swelling	1.2 cm, single mass	N/A	N/A	CR	12 m, NRM
Wang et al. (12)	1	28, F	Left leg	No	Multiple, firm, masses	Myxoid background, no giant cells or mitotic figures present	(+) Vimentin, SMA (–) Ketatin, S100, desmin	CR	N/A
Chi et al. (13)	1	20, F	Upper lip	No	0.5 cm, single, firm, mobile	Giant cells and mitotic figures (11/10 HPF) present	(+) SMA (–) S100	CR	24 months NRM
Reiser et al. (14)	1	58, F	Right cheek	Local swelling	1.7 cm, single	Focally myxoid and highly vascular background, no giant cells present, rare mitotic figures	(+) SMA, (–) S100, Desmin, EMA	CR	12 months NRM
Seo et al. (15)	1	26, M	Lower Lip	No	1 cm, single	Fibrous background, giant cells present with no mitotic figures	(+) SMA (–) CD31, CD34, desmin, S-100	CR	2 months NRM
Zheng et al. (16)	1	21, F	Flank	No	0.5 cm, single	Fibrous background, giant cells present with no mitotic figures	(+) SMA, FMSA (–) S100	CR	N/A
Min et al. (17)	1	29, F	Left common femoral vein	Pain swelling	4.5 cm, single	Spindle cells with mild nuclear atypia	(+) SMA, CD34 (–) Desmin, Ki67 (7%)	CR	3 months NRM
Lee et al. (18)	1	41, F	Left common Femoral vein	Swelling	3.8 cm, single	Fibrous background, present with no mitotic figures.	(+) SMA (–) S100	SRIV	48 m, NRM
Kuklani et al. (19)	2	25, F	Tongue and mouth	Swelling	Multiple, firm	Focally myxoid and highly vascular background, no giant cells present, rare mitotic figures	(+) SMA, MSA. (–) C-kit, DOG1, S100, GFAP Desmin, Ki-67 (<5%)	CR	12 months NRM
		26, M	Tongue	No	1.0 cm, single	Fibrous background, present with no mitotic figures.	(+) SMA, vimentin, CD34 (–) Desmin, S100. Ki-67 (<10%)	CR	5 months NRM
Takahashi et al. (20)	1	30, F	Right inguinal region	Pain	2.0 cm, single	Fibrous background, present with no mitotic figures.	(+) SMA, (–) Desmin, S100	CR	11 months NRM
Kang et al. (21)	1	44, F	Left clavicular intravenous	Pain	4.5 cm, single	Fibrous background, giant cells present with no mitotic figures	(+) SMA, (–) S100	CR	3 months NRM
Bartu and Dundr (22)	1	61, F	Ascending aorta	No	Single	Fibrous background present with no mitotic figures.	(–) SMA, desmin, S100 CD34, CD31, Ki-67 >25%	CR	12 months NRM
Ding and Jiang (23)	1	42, F	Left common femoral venous	Pain swelling	1.5 cm, single	N/A	(+) SMA, (–) S100, MYH9-USP6 rearrangement	CR	N/A
Le et al. (24)	1	23, F	Left common femoral vein	Pain swelling	Single, tender	Fibrous background, giant cells present with no mitotic figures	(+) SMA (–) S100	CR	24 months NRM
Li et al. (25)	1	39, F	Left common femoral vein	Pain swelling	Single	Myxoid background no giant cells present rare mitotic figures	(+) SMA (–) S100, desmin, FLI-1, ALK, CD34	CR	6 months, NRM
Pan et al. (26)	1	27, M	Left common femoral vein	Pain swelling	4.0 cm, Single	N/A	(+) SMA, vimentin (–) Desmin, S100	CR	14 months, NRM

* *Size in greatest dimension.*

*EMA, epithelial membrane antigen; HPF, high power field; LMWK, low molecular weight keratin; N/A, not applicable; SMA, smooth muscle actin; MSA, muscle-specific actin; FMSA, focal muscle specific actin; CR, complete resection; SRIV, Segmental excision of involved vein; NRM, no recurrence and metastasis; FLI-1, Friend leukemia virus integration-1*.

It can develop in almost any site and is most commonly encountered in the head and neck, followed by the upper extremities, lower extremities, and trunk (1, 18). There is no significant difference in the incidence of IVF between genders (5, 13, 19). Compared with ordinary NF, IVF was originally reported to affect the adolescent and young adult population. Most patients are between the ages of 20 and 40 years (17, 19). The mass can present as single or multinodular (17). Clinical symptoms usually manifest as a solitary nodule located subcutaneously or within muscular tissue. The size of the lesions range from 0.6 to 5 cm, and the course of the disease was 2 weeks−8 years (16). Other features manifested as with or without pain/tenderness, slow growing, mobility, and regional discomfort (20). Cases affecting the large veins often present symptoms of venous thrombosis. IVF is always misdiagnosed with thrombosis as was the case in our patient. There are no established imaging criteria for an accurate diagnosis of IVF. It is possible that high-resolution ultrasound accompanied by sensitive blood flow signals may distinguish the lesion from the thrombosis. The gross appearance is a non-encapsulated nodular mass with a firm-to-soft or gelatinous consistency. They may appear poorly circumscribed, and infiltrating masses and vascular invasion may be recognized. When the lesion was predominantly intravascular growth, it can form a serpentine or plexiform configuration (13).

Microscopically, IVF is mainly composed of a proliferation of plump spindle cells inside the lumen or associated with the wall of arteries and veins of all sizes, arranged in short intersecting fascicles, a storiform pattern, or a unregular manner. The lesions could appear as intraluminal or extraluminal growth with or without infiltration of surrounding connective tissue and infiltrative borders with entrapment of skeletal muscle observed in some cases. The background stroma was dense or myxoid and frequently showed a well-developed, slit-like capillary network. Scattered lymphocytes, red blood cells, and inflammatory cells often were seen in the background. Multinucleated giant cells were often present. Mitosis sometimes occurred, but no abnormal mitotic figures were found.

Immunohistochemistry studies often indicate that the spindle cells were positive for vimentin and SMA, negative for keratin, S100 protein, desmin, CD31, CD34, and c-kit, which confirmed the myofibroblastic differentiation seen in our patient (13, 19). A fusion gene (MYH9-USP6) caused by the chromosomal translocation *t*(17, 22) was detected in our case. The fusion gene (MYH9-USP6) was detected in 92% of NF and found to be highly sensitive and specific, favoring a neoplastic nature (27).

Due to the feature of infiltrative growth similar to a sarcoma (16), IVF also needs to be added to the differential diagnoses such as leiomyomatosis, hemangioendothelioma, fibroblastoma, myofibroblastoma, and leiomyosarcoma. Combined with the epidemiology, clinical symptoms, gross appearance, and microscopic and immunohistochemistry of IVF, it is not difficult to distinguish it from other tumors, especially microscopically and with immunohistochemistry. Surgical resection is the main method to treat IVF, and perioperative management is the guarantee of good surgical efficacy. It is very important to fully evaluate the nodule before surgery, including CTA and ultrasound contrast. According to current literature reports, there is no preoperative pathological puncture for IVF. The nodules should be completely removed during the operation, including the invaded vascular wall. As to whether the invaded vasculature needs to be reconstructed, it needs to be comprehensively evaluated based on the diameter, location, and scope of the invaded vasculature. Patients that are revascularized should be anticoagulated after surgery to avoid thrombosis. In general, surgical resection is the main treatment of IVF and the reoccurrence rate of IVF is <1–2% (12, 13).

## Conclusion

IVF arising in the jugular vein is extremely uncommon. IVF is very easily misdiagnosed as sarcoma, thrombosis, or other low-grade malignant mesenchymal neoplasms. Combined with the epidemiology, clinical symptoms, gross appearance, microscopic and immunohistochemistry of IVF, it is not difficult to distinguish it from other tumors, especially microscopically and with immunohistochemistry.

## Data Availability Statement

The original contributions presented in the study are included in the article/supplementary material, further inquiries can be directed to the corresponding authors.

## Ethics Statement

Written informed consent was obtained from the individual(s) for the publication of any potentially identifiable images or data included in this article.

## Author Contributions

GC and CC were wrote the manuscript and were assistant in surgery. XW was involved in editing the manuscript. BH was chief operating surgeon. All authors contributed to the article and approved the submitted version.

## Conflict of Interest

The authors declare that the research was conducted in the absence of any commercial or financial relationships that could be construed as a potential conflict of interest.

## Publisher's Note

All claims expressed in this article are solely those of the authors and do not necessarily represent those of their affiliated organizations, or those of the publisher, the editors and the reviewers. Any product that may be evaluated in this article, or claim that may be made by its manufacturer, is not guaranteed or endorsed by the publisher.
